# The Effect of Including eHealth in Dietary Interventions for Patients with Type 2 Diabetes with Overweight or Obesity: A Systematic Review

**DOI:** 10.3390/nu15173776

**Published:** 2023-08-29

**Authors:** Karlijn A. M. Geurts, Sandra Woodcock-Nekeman, Mitchell Hummel, Carmen A. W. Dietvorst, Elisabeth F. C. van Rossum, Kirsten A. Berk

**Affiliations:** 1 Department of Internal Medicine, Division of Dietetics, Erasmus MC, University Medical Centre Rotterdam, Doctor Molewaterplein 40, 3015 GD Rotterdam, The Netherlands; k.geurts@erasmusmc.nl (K.A.M.G.); s.woodcock-nekeman@erasmusmc.nl (S.W.-N.); c.dietvorst@erasmusmc.nl (C.A.W.D.); 2 Department of Internal Medicine, Division of Endocrinology, Erasmus MC, University Medical Centre Rotterdam, 3015 GD Rotterdam, The Netherlands; e.vanrossum@erasmusmc.nl; 3 Obesity Center CGG, Erasmus MC, University Medical Centre Rotterdam, 3015 GD Rotterdam, The Netherlands

**Keywords:** eHealth, type 2 diabetes, dietary intervention, diet, overweight, obesity

## Abstract

eHealth has a growing impact on the delivery of healthcare, making health systems more efficient. This study examined the effect of dietary interventions using eHealth compared to face-to-face contact in patients with (pre-) type 2 diabetes (T2D) and who are overweight/obese. Literature databases were searched upon November 2022. Inclusion criteria: randomized controlled trial; duration ≥ 6 months; involving dietary interventions; performed in adults with (pre-) T2D and who are overweight/obese; using eHealth compared to face-to-face contact; and report outcomes on weight loss, glycemic regulation, and/or cost-effectiveness. Selection of articles was performed manually and using ASReviewLab. Fifteen studies were included for data extraction, investigating a wide variety of eHealth interventions. Seven studies reporting on weight loss showed a significant between-group difference (−1.18 to −5.5 kg); five studies showed a trend in favor of the eHealth programs. Eleven studies reported on HbA1c; three found a significant between-group difference (−0.23 to −0.70%) in favor of the eHealth programs and six studies showed non-significant improvements. Interaction with healthcare professionals led to better results of the dietary interventions. Two studies reported incomplete data on cost-effectiveness. In conclusion, eHealth shows better results of dietary interventions in (pre-) T2D patients compared to face-to-face, especially when combined with interaction with healthcare professionals.

## 1. Introduction

Electronic Health (eHealth) has a growing impact on the delivery of healthcare around the world today, making healthcare systems more efficient and more responsive to people’s needs and expectations [1]. The World Health Organization (WHO) has formulated a global strategy on digital health were they state their vision: “improving health for everyone, everywhere by accelerating the development and adoption of appropriate, accessible, affordable, scalable and sustainable person-centric digital health solutions”. For this to be successful digital health needs to be accessible and enhance the efficiency of healthcare systems in delivering quality and affordable care [2].

Despite increased interest and application of eHealth in recent years, a uniform definition is still missing. One of the definitions of eHealth by Eysenbach et al. states that “eHealth refers to health services and information delivered or enhanced through the Internet and related technologies. In a broader sense, the term characterizes not only a technical development, but also a state-of-mind, a way of thinking, an attitude, and a commitment for networked, global thinking, to improve healthcare locally, regionally, and worldwide by using information and communication technology” [3].

The eHealth monitor in The Netherlands (2019) revealed that more than half of the participating healthcare professionals and more than a third of chronically ill patients believe that applications (apps) and websites can give more insights into patients’ health, particularly when these apps are specifically tailored to the patients’ personal health or treatment needs [4]. When eHealth is well embedded in patient care, it can reduce the workload of healthcare personnel [5]. Especially in rapidly growing patient populations, such as the population of people with obesity-related type 2 diabetes, eHealth can be an efficient method of reaching more patients with the same number of care professionals. Worldwide, 415 million people are living with type 2 diabetes, which is expected to rise to half a billion people in 2040 [6]. Only a handful of reviews and meta-analyses have been conducted on the effect of eHealth on diabetes self-management, mainly focusing on the improvement in glycemic regulation. These reviews show a clinically relevant reduction of hemoglobin type A1c (HbA1c) in the eHealth group compared to the usual care group [7,8]. However, interventions specifically targeting diet were not included. Dietary interventions have proven to be effective in reducing weight and improving glycemic regulation, cholesterol levels, and blood pressure in T2D [7]. Face-to-face dietary interventions are often lengthy and time-consuming and should be guided by specialized dietitians. For this very reason, in this ever-growing patient population, it is a huge challenge to be able to provide guidance to all patients who need it. The use of eHealth, whether combined with face-to-face contact or not, can potentially provide a solution.

To our knowledge, no systematic review has been carried out investigating eHealth interventions in the dietary treatment of type 2 diabetes patients who are overweight or obese, on weight loss, on glycemic regulation, and/or its cost-effectiveness. Therefore, the purpose of this systematic review is to provide a general overview of the effect of dietary interventions using eHealth only or in combination with face-to-face interventions, compared to face-to-face only in patients with type 2 diabetes and are overweight or obese.

## 2. Methods

This review protocol was registered in the International Prospective Register of Systematic Reviews (PROSPERO) with registration number CRD42021264235. Details and justification of any changes during the review process were added to the registration in PROSPERO to minimize potential bias.

This systematic review was performed in accordance with the Preferred Reporting Items for Systematic Reviews and Meta-Analysis (PRISMA) guidelines [9]. In this systematic review, telephone calls were not considered eHealth, because they are part of routine care.

We used the following definition for eHealth: “health services and information delivered or enhanced through the Internet and related technologies”. This is based on the definition of Eysenbach et al. [3].

In this review, patients with diabetes type 2 and prediabetes were included, defined according to the guidelines of American Diabetes Association [10].

### 2.1. Search Strategy

Embase, Medline, Web of science core collection, PsycINFO Ovid, and Cochrane CENTRAL register of trials were searched, with no publication date restriction, published in English. A systematic search was performed for eligible studies published up to 30 January 2020. Too much time had elapsed between the search and writing of this review. Therefore, a second search was performed including studies published up to 17 November 2022. The search terms for both were ‘diabetes’ AND ‘obesity’ OR ‘weight loss program OR diet therapy OR low-calorie diet’ AND ‘group education OR support group OR telehealth OR lifestyle class’. Full search strings can be found in the Supplemental Material.

### 2.2. Inclusion and Exclusion Criteria

Studies were included if they met the following criteria:

- Randomized controlled trial;
- ≥6 months in duration;
- Involving a dietary intervention (defined as an intervention on diet more than just diabetes guidelines);
- Performed in overweight or obese adults (defined as a body mass index (BMI) ≥ 25 kg/m^2^ in a Caucasian population or a BMI ≥ 23 kg/m^2^ in an Asian population) with type 2 diabetes or prediabetes;
- Using face-to-face combined with eHealth (blended) or eHealth only, compared to a control intervention;
- Report an outcome on weight loss, glycemic regulation and/or cost effectiveness.

The following study population characteristics were excluded during screening: pregnant or lactating women, women with gestational diabetes (in the recent past), patients with metabolic diseases other than type 2 diabetes, and severe mental illness or carcinomas of any kind.

### 2.3. Study Selection

**Figure 1:**
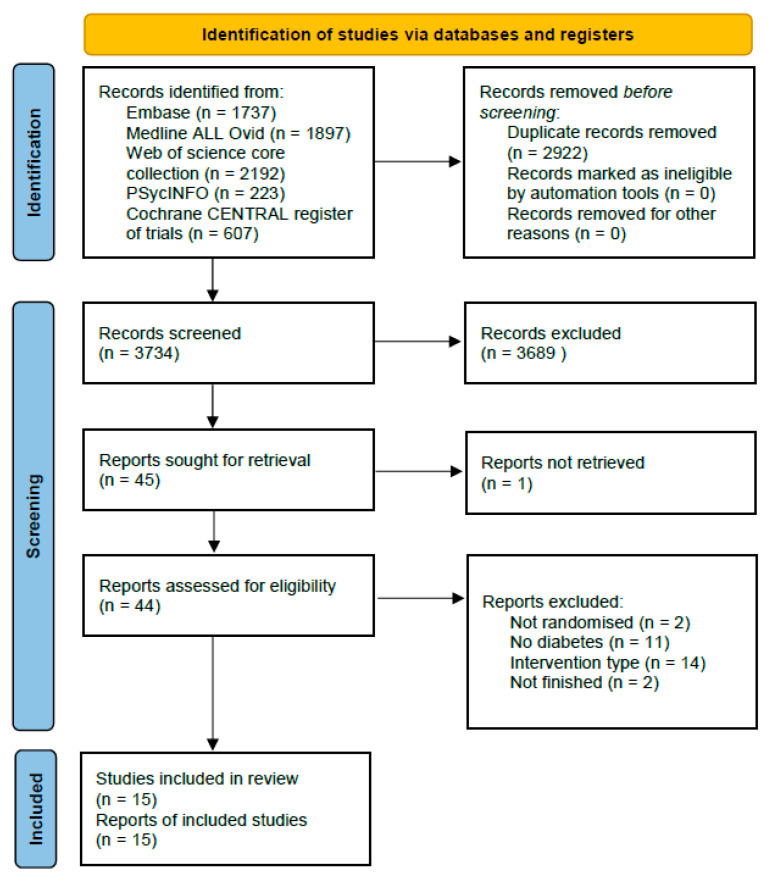
PRISMA flow diagram.

Records resulting from the search were screened by reviewing the title and abstract based on the inclusion and exclusion criteria. One reviewer (KAMG) prepared the initial dataset by removing all duplicates. The first reviewer (KAMG) screened manually using EndNote (version X9, Clarivate Analytics©, London, UK). Next, KAMG selected ten relevant and ten irrelevant articles, which were used by an artificial intelligence (AI) model to select the relevant output. The second reviewer (MH) imported the dataset into ASReview Lab (Version v0.17rc0, ASReview©, Utrecht, The Netherlands), a system based on artificial intelligence with default settings for classifiers and file extraction. The second reviewer stopped screening after hundred consecutive irrelevant articles [11]. Selected studies (manually and using AI) based on title and abstract were compared, and any disagreements about inclusion or exclusion were discussed until consensus was reached. A third reviewer (KACB) was asked to join the discussion, when no consensus could be reached between the two reviewers, until there was a final decision. Afterwards, both reviewers performed a full-text screening to make a final selection. Here, the discussion process was repeated. This screening process was repeated after the second updated literature search, with as second reviewer SWN instead of MH. Screening in ASReview Lab this time was stopped after fifty consecutive irrelevant articles, because of the smaller number of records, with a similar percentage of records screened [11]. The study selection process, including reasons for exclusion of records, is summarized in a PRISMA flow diagram and presented in Figure 1.

### 2.4. Data Collection Process

Two reviewers extracted the data from the selected studies independently to a Microsoft Excel 2016 spreadsheet. Afterwards the Excel spreadsheets were compared and a final dataset was formed. When there were inconsistencies, which could not be solved by the article, original authors were contacted. Data were extracted on the following:

- Type of dietary intervention that was used;
- Type of eHealth that was used;
- Demographic variables of the study population;
- Duration of the study;
- The outcome variables weight loss and/or costs of the treatment;
- Other variables, when available (HbA1c and body composition measurements).

The original authors were contacted if information was missing.

### 2.5. Risk of Bias in Individual Studies

The Risk of Bias 2 tool (RoB2 tool, 2021) of Cochrane (The Cochrane Collaboration) [12] was used by all reviewers independently to assess the risk of bias at the outcome level. The RoB2 tool has three levels, which are described in Table 1. For the assessment an Excel tool was used, which was provided by Cochrane. Risk of bias was assessed based on weight loss outcomes or HbA1c; fasting glucose was only used when weight loss and HbA1c were unavailable. Only outcomes measured pre- and post-intervention were used.

### 2.6. Data Synthesis and Analysis

No meta-analysis was undertaken, because of the wide variety of diet interventions and eHealth applications found. Therefore, study characteristics were summarized in a table and discussed. We converted all weight loss outcomes to the same unit; also, HbA1c and fasting blood glucose were converted for comparison: weight was noted in kilograms and fasting glucose in mg/dl. When possible, a qualitative synthesis was conducted.

## 3. Results

### 3.1. Description of Studies

Figure 1 shows a flow diagram of the search and selection process. The included studies were published between 2011 and 2022. The first search selected 2614 articles excluding duplicates and one article was found by snowballing [13]. Only eight articles fulfilled all our inclusion criteria and were included in this review [13,14,15,16,17,18,19,20]. The second search selected 1120 articles excluding duplicates. Of this search, only seven articles fulfilled all our inclusion criteria and were included in this review [21,22,23,24,25,26,27]. Table 2 shows the characteristics of the fifteen included studies. Two out of the fifteen studies included were pilot randomized controlled trials (RCT) with a small number of participants [16,20]. All studies had a superiority design.

### 3.2. Quality Assessment of Included Studies

The quality of all fifteen included studies was assessed using the Risk of Bias 2 Tool [12], as shown in Table 3. All fifteen studies included in this review used random sequence generation. Blinding of participants was, because of the nature of the intervention, not possible. Blinding of personnel measuring outcome assessment was only used by two studies [18,21]. Overall, the risk of bias of the included studies was low.

### 3.3. Study Population

The included studies examined a total of 3103 participants. Ten studies took place in North America (seven in the United States of America [15,18,20,21,22,23,24], two in Canada [14,17], and one in Mexico [19]), three in Asia (one in Saudi Arabia [25], one in Singapore [26], and one in China [27]), and two in Europe (one in the United Kingdom [16] and one in Italy [13]). Most participants were older than 45 years. Eight studies included patients with type 2 diabetes, of which three studies included patients with a type 2 diabetes duration of >5 years [17,19,26]. The other five studies included patients who were diagnosed with type 2 diabetes six months to four years before the study [13,16,20,23,27]. Seven studies included patients with prediabetes [14,15,18,21,22,24,25]. Two studies only included women [23,25] and one study only included men [16,17,25].

### 3.4. Intervention Characteristics

Follow-up duration varied between 6 and 21 months. Six of the included studies used the diabetes prevention program (DPP) [28] as the basis for their dietary intervention [15,18,20,22,24]; the other studies used national dietary diabetes guidelines.

The types of eHealth used in the intervention group were the use of an interactive website [13,16,18,19,21,23], a pedometer [14,18,22], a smartphone application [13,20,22,23,24,25,26,27], text messaging [15], and email [17].

The comparisons used as control groups also showed a wide variety, ranging from usual care by a diabetes team [14,15,16,18,20,24,27], one (group) meeting with or without printed information [19,21,22,23,25,26], a 16-week group program [17], or one-month admission [13].

### 3.5. Weight Loss

Weight loss was reported in fourteen studies. Some reported percentage weight lost, others reported kilograms or pounds or both. Seven studies showed a significant between-group difference in weight loss in favor of the eHealth group, ranging from 1.18 to 5.5 kg extra weight loss compared to the control group [14,15,21,22,24,25,26]. Five of these studies used any form of interaction in their eHealth intervention. In another five studies a greater weight loss was found in the eHealth group (with interaction), but these were non-significant findings [13,16,19,20,23]. Two studies found the opposite, namely a significantly greater weight loss in the control group [17,18]; both these studies had no interaction in the eHealth intervention group.

### 3.6. Costs

Only two studies reported an outcome on costs of the intervention. Unfortunately, only the costs for the intervention group were reported, so no comparisons could be made. Dawes et al. [14] reported the mean costs of the intervention per participant, namely CAD 144.

Fischer et al. [15] also reported extra costs on top of usual care for the intervention group, which was GBP 22,114 in total over the 1-year intervention period. With 79 participants in the intervention group this equals GBP 280 per participant. No comparisons were made between the costs of the control and intervention groups, and cost-effectiveness analyses were not performed.

### 3.7. HbA1c

Eleven studies reported HbA1c. Three found a significant between-group difference with a decrease of 0.23 to 0.70% in favor of the eHealth group [21,22,26]. All used interaction in their eHealth intervention group. Non-significant differences were found in six studies in favor of the eHealth group [14,15,19,23,24,27] of which five used a form of interaction in the eHealth intervention group [16]. Wang et al. did not find a difference even though the eHealth group interacted with a healthcare professional, while Lutes et al. found a non-significant increase in HbA1c in the eHealth group without any form of interaction [15,23].

### 3.8. Fasting Blood Glucose

Six studies analyzed fasting glucose levels of which four found a significant between-group difference with a decrease in the eHealth group between 2.7 to 18.1 mg/dL [18,19,21,26]. Three of these studies used a form of interaction in the eHealth intervention group [16,24,26]. Two studies found a non-significant decrease in fasting blood glucose in favor of the eHealth group with interaction with a healthcare professional [22,27].

### 3.9. BMI

Eight studies reported on BMI. Six showed a significant between-group difference in BMI with a decrease of 0.9 to 3.77 kg/m^2^ in favor of the eHealth group [14,18,21,24,26,27]. Two studies showed a trend towards a larger decrease in BMI in the eHealth group, both using a form of interaction [16,19].

### 3.10. Waist Circumference

Five studies reported on waist circumference, of which four studies conducted an eHealth intervention with interaction with a healthcare professional [14,16,22,24]. Three studies showed a significant between-group difference in waist circumference with a decrease between 3 to 4.9 cm in favor of the eHealth group [14,18,21]. Velazquez et al. found a reduction in waist circumference; however, this was not significant [24]. Haste et al. reported a trend towards a larger decrease in waist circumference in the eHealth group, but significance was not stated [14].

### 3.11. Fat Mass

One study reported on percentage fat mass and showed a non-significant between-group difference of −0.69% in favor of the eHealth intervention group [19].

## 4. Discussion

The aim of this systematic review was to provide a general overview of the effect and/or cost-effectiveness of dietary interventions using eHealth or a combination of eHealth and face-to-face contact in comparison with control dietary interventions in patients with (pre-) type 2 diabetes who were overweight or obese.

From this systematic review we can conclude that eHealth is a beneficial tool for healthcare professionals and overweight or obese patients with type 2 diabetes to help improve weight and glucose regulation: Fourteen studies reported on weight loss of which almost all showed a significantly greater decrease or positive trend in favor of the eHealth group. Three out of eleven studies reporting on glucose regulation found a significant between-group difference of HbA1c in favor of the eHealth group, and another six studies showed a positive trend. Interaction with a healthcare professional led to better results of the eHealth dietary interventions. No conclusion could be drawn on the cost-effectiveness of eHealth interventions as only two studies reported data on costs. A comparison could not be made because costs of the control group were not available.

The fifteen included articles showed a large variation in effect on mean weight reduction and HbA1c. An explanation for the differences found in results could be that in some studies during the dietary intervention medication use was reduced due to positive blood glucose levels. As a result, no significant differences can be found between HbA1c levels before and after the intervention while weight loss and other positive health effects were achieved. Another reason that may affect the results of eHealth in these studies is the variation of eHealth interventions used, varying from sending an email once a week, to an interactive web based program and app. Moreover, the eHealth intervention groups in most studies had more contact hours with a healthcare professional compared to the control groups. A systematic review of Chrvala et al. suggested that contact hours exceeding 10 are more often associated with a significant reduction of HbA1c in T2D patients [29]. Therefore, the question is whether there is an effect due to the intervention or the number of contact moments. However, in this review we see a better outcome if eHealth is combined with face-to-face contact or interaction with a healthcare professional, in concordance with the literature [14,15,18]. When eHealth is used without interaction with a healthcare professional, we see the opposite: weight loss and glucose regulation were significantly worse in comparison with the group who had face-to-face contact with a healthcare professional [17,18]. This has also been demonstrated in non-nutrition-related eHealth interventions with T2D patients. Participants who used eHealth in combination with any form of interaction with a healthcare professional had significant improvements in outcomes such as weight, waist circumference, glucose regulation, diabetes self-efficacy, and depressive symptoms [30,31,32]. This suggests that regardless of the type of intervention, applying blended care is beneficial for people with T2D.

In a systematic review and meta-analysis on eHealth interventions in overweight and obese patients without T2D, Hutchesson et al. reported that there was a significantly greater weight loss in the eHealth group compared to the control group (MD −2.70 [−3.33, −2.08], *p* < 0.00001; MD −1.40 [−1.98, −0.82], *p* < 0.0001). Moreover, compared to eHealth only, a significantly greater weight loss was found in the intervention subgroup where eHealth was combined with interaction with a healthcare professional [33]. Similarly, Puigdomenech et al. from a systematic review on the effectiveness of weight control in obese patients found that interventions including face-to-face elements produced significantly better outcome on weight loss [34]. This is in line with our findings, showing that eHealth interventions using some form of interaction like group sessions, web-based consultations, phone calls, or chat lines lead to positive effects on weight loss, whereas eHealth interventions without interaction had no or even negative results.

In contrast with the findings of Hutchesson et al. [33] in this review, there are also a few studies that show a better result in weight loss where the eHealth group does not have face-to-face contact. Here, interaction with healthcare professionals in the effective eHealth interventions was not face-to-face but instead occurred via personalized feedback within the eHealth intervention. This type of interaction was also found effective in the review of Sherrington et al. [35]. Thus, when implementing blended care in practice, the form of interaction does not matter for the outcome. It can be tailored to the needs of the patient and healthcare professional.

The message of this systematic review that blended care seems more effective than face-to-face contact only in the context of dietary interventions is an important one, as the COVID-19 pandemic has shown that there is a need for alternative ways to meet with your healthcare professional. Even after the pandemic, the call for more efficient remote care to help more patients remains, including those who are unable to come to healthcare facilities (often). The studies included here were executed in several continents, which makes the results easily applicable to a broad population.

This systematic review has some limitations. First, the eHealth definition of Eysenbach et al. was used for the selection of articles. However, this definition is one of many used in scientific articles, which makes it challenging to compare the results with other reviews on this topic [36,37]. In addition, the different definitions of eHealth include a broad spectrum of eHealth methods. In this review, we also see a large variety of methods of eHealth used, which makes it difficult to conclude on the effectiveness of eHealth in general. Second, two studies included were pilot studies, and one study was an interim analysis, which makes it more likely that these studies were underpowered. In addition, because weight was not the primary outcome in all included studies, the power of these findings can be questioned [13,15,17]. Third, all the included studies had a superiority design, which means they aimed to find a significant better outcome in the intervention group in comparison to the control group. It can be debated, however, whether a superiority trial is the best design to assess the effectiveness of eHealth. A non-inferiority design may prove to be a better fit if the research is focused on important benefits (costs, retention, and usages) when using eHealth as opposed to an active control treatment [38]. In this design finding, an equal result could lead to the replacement of the control intervention with the eHealth intervention without loss of effectivity. In addition, we included only RCTs and excluded observational studies from this review. Although observational studies could offer more data and useful insights, the quality of the research is lower. For example, there is no insight into the equality of the groups and the question is whether you can say anything about the results in that case. Fourth, very few included reviews had inclusion criteria related to participants’ socioeconomic status, while this may affect the results. Therefore, the generalizability of the reported findings could be challenging. However, the use of eHealth (websites, apps) has skyrocketed in recent years among all walks of life, regardless of age or socioeconomic background [39]. Although using eHealth requires some skills, statistics show that over 70% of the Dutch population up to 75 years of age with various educational backgrounds use some form of eHealth almost daily. Given that the average age at which T2D is diagnosed is 61 years, applying eHealth in this population should present no limitations [40]. Fifth, the included studies are relatively short-term interventions making it difficult to draw conclusions about long-term effects that depend on patient compliance. Further research is needed to determine whether long-term outcomes are comparable to those in this systematic review.

## 5. Conclusions

This systematic review indicates an important place for eHealth in the dietary treatment of patients with (pre-) type 2 diabetes who were overweight or obese. Evidence suggests that including some form of interaction with a healthcare professional is positive for the effectiveness of the eHealth intervention, and can contribute to motivating the patients.

These results suggest that eHealth is a tool that can be used in current practice, but additional research is needed for integration into guidelines or for policy makers.

This additional research should focus on the cost-effectiveness of eHealth interventions compared with existing effective face-to-face interventions. In addition, research is needed to determine which eHealth interventions or components of eHealth are most effective in improving long-term dietary interventions for weight loss and glycemic control in patients with type 2 diabetes and obesity.

## Figures and Tables

**Table 1:** Risk-of-bias judgement for a specific outcome.

Overall Risk-of-Bias Judgement	Criteria
Low risk of bias	The study is judged to be at low risk of bias for all domains for this result.
Some concerns	The study is judged to raise some concerns in at least one domain for this result but not to be at high risk of bias for any domain.
High risk of bias	The study is judged to be at high risk of bias in at least one domain for this result.
	Or
	The study is judged to have some concerns for multiple domains in a way that substantially lowers confidence in the result.

**Table 2:** Study characteristics.

Study			Participant Characteristics			Results		
First Author; Year; Duration; Design	Intervention Groups *	Sample	Age (y)	Male (%)	BMI (kg/m^2^)	Anthropometric Measurements		Other Measurements
Al Hamdan et al., 2021 [25] 6 months RCT	[1] Control group	253 overweight or obese female with prediabetes	[1] 51 ± 7.1	[A] 0	[1] 31.6 ± 5.8	*Weight change (kg)*	*WC (cm)*	*HbA1c (%)*
						[1] −0.2	[1] +0.2	[1] −0.3 **
						[2] −5.8 **	[2] −1.1 **	[2] −0.7 **
						[3] −1.3 **	[3] −3.5 **	[3] −0.5 **
						[D] **	[D] **	[D] *p* = 0.33
	[2] eHealth education via app		[2] 44 ± 8.1		[2] 30.0 ± 5.1	*BMI (kg/m^2^)*		
	[3] Face-to-face—group education		[3] 43 ± 12.2		[3] 34.8 ± 9.0	[1] −0.1		
						[2] −0.6 **		
						[3] −2.1 **		
						[D] **		
Block et al., 2015 [21] 6 months RCT	[1] Control group	340 overweight or obese male and female with prediabetes	[1] 55 (9.1)	[1] 69	[1] 31.2 (4.3)	*Weight change (kg)*	*WC (cm)*	*HbA1c (%)*
						[1] −1.26 (−1.27, −1.26)	[1] −2.22 (−2.36, −2.09)	[1] −0.18 (−0.19, −0.16)
						[2] −3.26 (−3.26, −3.25) **	[2] −4.56 (−4.69, −4.43) **	[2] −0.26 (−0.27, −0.24) **
	[2] eHealth via web based program		[2] 55 (8.8)	[2] 68	[2] 31.1 (4.5)	*Weight change (%)*		*Fasting glucose (mmol/L)*
						[1] −1.32 (–1.36, −1.28)		[1] −0.12 (−0.15, −0.10)
						[2] −3.60 (−3.63, −3.57) **		[2] −0.41 (−0.44, −0.38) **
						*BMI (kg/m^2^)*		
						[1] −0.39 (−0.39, −0.38)		
						[2] −1.05 (−1.06, −1.05) **		
Castelnuovo et al., 2011 [13] 12 months; RCT	[1] Control group	34 obese male and female with type 2 diabetes	[1] 49 (46–57.5)	[1] 69 [2] 35	-	*Weight change (%)*		
						[1] −4.1 (−15.3 to 3)		
	[2] eHealth via web based program		[2] 54 (49–60)			[2] −6.2 (−10.6 to 16.7)		
Dawes et al., 2015, [14] 6 months; RCT	[1] Control group	59 overweight or obese male and female with prediabetes	47% aged 35–64,	[A] 51	[A] 29.1 ± 5.9	*Weight change (kg)*	*WC (cm)*	*Fasting glucose (mg/dL)*
						[1] −0.3 ± 1.8	[1] −1 ± 5	[1] −0.54 ± 8.47
						[2] −3.4 ± 3.1	[2] −4 ± 5	[2] −3.96 ± 7.39
						[D] −3.2 (−4.6 to −1.7) #	[D] −3 (−5.7 to −0.3) #	[D] −3.24 (−7.57 to 1.08) #
	[2] eHealth with pedometer + telephone calls		53% aged ≥ 65			*BMI (kg/m^2^)*		*HbA1c (%)*
						[1] 0.0 ± 0.7		[1] 0.03 ± 0.24 [2] −0.07 ± 0.21
						[2] −1.2 ± 1.1		[D] −0.10 (−0.23 to 0.03) #
						[D] −1.2 (−1.7 to −0.7) #		*Mean cost per participant (C$)* [2] 144.
Fischer et al., 2016 [15] 12 months; RCT	[1] Control group	157 overweight or obese male and female with prediabetes	[1] 45 ± 10.6	[1] 19	-	*Weight change (kg)*		*HbA1c (%)*
						[1] −0.25 (−1.23 to 0.73)		[1] 0.19 (−0.1 to 0.5)
						[2] −1.18 (−2.50 to 0.09)		[2] −0.09 (−0.2 to 0.0)
	[2] eHealth by text messages		[2] 48 ± 12.4	[2] 30		[D] −0.95 (−2.54 to 0.64)		[D] −0.29 (−0.58 to 0.01)
								*Total intervention program cost ($)*
								[2] 22,113.61
Haste et al., 2017 [16] 12 months; pilot RCT	[1] Control group	61 obese male with type 2 diabetes	[1] 61 {54.5, 66.8}	[A] 100	[1] 34.6 ± 3.0	*Weight change (kg)*	*WC (cm)*	
						[1] −2.8 ± 4.4	[1] –2.0 (–3.8 to –1)	
						[2] −5.4 ± 5.9 #	[2] –3.5 (–7 to –1.3) #	
	[2] eHealth by web based program		[2] 58 {50, 67.5}		[2] 33.9 ± 2.6	*BMI (kg/m^2^)*		
						[1] −0.9 ± 1.4		
						[2] −1.3 ± 2.0 #		
Katula et al., 2022 [22] 12 months single blind RCT	[1] Enhanced control group	599 overweight or obese male and female with prediabetes	[1] 56 (12.6)	[1] 39	[1] 36.1 (6.6)	*Weight change (kg)*		*HbA1c (%)*
						[1] −2.18 (2.97, 1.39)		[1] −0.16 (−0.19, −0.12)
						[2] −5.52 (6.30, 4.75)		[2] −0.23 (−0.26, −0.20)
						[D] 3.34 (4.39, 2.29) **		[D] −0.08 (−0.12, −0.03) **
	[2] eHealth by web based program and pedometers		[2] 55 (12.9)	[2] 39	[2] 35.8 (6.1)	*Weight change (%)*		
						[1] 2.09 (2.82, 1.37)		[1] 1.70 (2.07, 1.33),
						[2] 5.49 (6.20, 4.78)		[2] 2.52 (2.89, 2.16),
						[D] 3.40 (4.36, 2.43) **		[D] 0.82 (1.32, 0.32) **
Lim et al., 2021 [26] 6 months Multi center RCT	[1] Control group	204 overweight or obese male and female with type 2 diabetes	[1] 51 (10.0)	[1] 63	[1] 30.9 (4.5)	*Weight change (kg)*		*HbA1c (%):*
						[1] −1.2 (3.6)		[1] −0.3 (1.0)
						[2] −3.6 (4.7)		[2] −0.7 (1.2)
						[D] −2.4 (−3.5 to −1.3) **		[D] −0.4 (−0.7 to −0.1)**
	[2] Control group + app		[2] 52 (9.4)	[2] 67	[2] 30.3 (4.0)	*Weight change (%)* [1] −1.4 (4.2), [2] −4.3 (5.4), [D] −2.9 (−4.2 to −1.6) **		*Fasting glucose (mg/dL)* [1] −1.8 (25.2)
						*BMI (kg/m^2^)*		
						[1] −0.4 (1.3)		
						[2] −1.3 (1.7)		[2] −14.4 (37.8)
						[D] −0.9 (−1.3 to −0.5) **		[D] −12.6 (−23.4 to −3.6) **
Lutes et al., 2017 [17] 12 months; RCT	[1] Face-to-face group education [2] eHealth by email	200 overweight or obese female with type 2 diabetes	[1] 53 ± 10.62	[A] 0	[1] 36.59 ± 7.48	*Weight change (kg)*		*HbA1c (%)*
			[2] 54 ± 9.84		[2] 38.80 ± 8.43	[1] −1.35 ± 6.22		[1] −0.29 ± 1.84
			[A] 53 ± 10.24		[A] 37.67 ± 8.02	[2] −0.39 ± 4.57 **		[2] 0.05 ± 1.61
Ma et al., 2013 [18] 15 months; RCT	[1] Control group	241 overweight or obese male and female with prediabetes or metabolic syndrome	[1] 53 ± 10.9	[A] 54	[1] 32.4 ± 6.3	*Weight change (kg)*	*WC (cm)*	*Fasting glucose (mg/dL)*
						[1] −2.4 (0.9)	[1] −2.2 (1.1)	[1] 0.2 (1.7)
						[2] −4.5 (0.9)	[2] −4.9 (1.0)	[2] −2.7 (1.6)
						[3] −6.3 (0.9)	[3] −5.8 (1.0)	[3] −4.2 (1.6)
						[D] **		
	[2] eHealth by Digital Versatile Disk (DVD) and pedometer		[2] 52 ± 9.9		[2] 31.7 ± 4.7	*Weight change (%)*		
						[1] −2.6 (0.9)		
						[2]−5.0 (0.9)		
						[3] −6.6 (0.9)		
	[3] Face-to-face group education		[3] 55 ± 11.0 [A] 53 ± 10.6		[3] 31.8 ± 5.1 [A] 32.0 ± 5.4	*BMI (kg/m^2^)*		
						[1] −0.9 (0.3)		
						[2]−1.6 (0.3)		
						[3] −2.2 (0.3)		
						[D] **		
St-Jules et al., 2022 [23] 6 months 2 × 2 factorial RCT	[1] Written information and telephone calls on education	256 overweight or obese male and female with type 2 diabetes	[1] 67 (9.0)	[1] 48	[1] 33.3 (4.5)	*Weight change (kg)* [1] −1.2 (4.3)		*HbA1c (%)* [1] −0.3 (1.1)
	[2] eHealth by video conferencing focused on education and a web based program		[2] 65 (10.0)	[2] 56	[2] 34.4 (5.5)	[2] −2.3 (3.4)		[2] −0.3 (0.9)
	[3] eHealth by video conferencing focused on education and behaviour		[3] 64 (9.0)	[3] 42	[3] 34.2 (5.7)	[3] −1.4 (3.1)		[3] −0.1 (1.0)
	[4] eHealth by video conferencing focused on education and behaviour and a web based program		[4] 64.0 (8.0)	[4] 53	[4] 33.2 (4.4)	[4] −2.7 (4.4)		[4] −0.3 (0.9)
Toro-Ramos et al., 2020 [24] 12 months; RCT	[1] enhanced control group	202 overweight or obese male and female with prediabetes	[1] 58 (12.5)	[1] 31	[1] 30.9 (7.2)	*Weight change (kg)*		*HbA1c (%)*
						[1] −0.09 (−1.30 to 1.11)		[1] −0.16 (−0.27 to −0.05) **
						[2] −2.22 (−3.31 to −1.13) **		[2] −0.23 (−0.32 to −0.14) **
						[D] −1.8 **		
	[2] eHealth by Noom web-based program and app		[2] 56 (13.6)	[2] 26	[2] 31.3 (6.4)	*Weight change (%)* [1] 0.33 (−1.06 to 1.72)		
						[2]−2.54 (−3.74 to −1.33) **		
						*BMI (kg/m^2^)* [1]−0.04 (−0.47 to 0.39)		
						[2]−0.88 (−1.31 to 0.44) **		
						[D] −0.58 **		
Velázquez-López et al., 2017 [19] 21 months; RCT	[1] Control group	351 overweight or obese male and female with type 2 diabetes	[1] 54 ± 8.8	[1] 34	[1] 30.4 ± 5.0	*Weight change (kg)*	*WC (cm)*	*Fasting glucose (mg/dL)*
						[1] −0.61 (−1.47 to 0.25)	[1] −4.32 (−5.59 to −3.04)	[1] −6.50 (−17.8 to −4.70)
						[2] −1.23 (−2.29 to −0.16)	[2] −5.50 (−6.89 to −4.12)	[2] −36.6 (−46.6 to −26.60)
						[D] −0.62 (−1.97 to 0.74)	[D] −1.19 (−3.06 to 0.68)	[D] −18.1 (−29.7 to −6.4) **
	[2] Usual care and eHealth by web-based program		[2] 55 ± 8.8	[2] 30	[2] 30.8 ± 5.9	*BMI (kg/m^2^)*	*Fat mass (%)*	*HbA1c (%)*
						[1] −0.07 (−0.39 to 0.25)	[1] −0.27 (−1.57 to 1.03)	[1] −1.33 (−1.65 to −1.01)
						[2] −0.42 (−0.86 to 0.01)	[2] −0.96 (−2.17 to 0.26)	[2] −1.48 (−1.91 to −1.04)
						[D] −0.36 (−0.89 to 0.18)	[D] −0.69 (−2.46 to 1.08)	[D] −0.11 (−0.70 to 0.48)
Wang et al., 2018 [20] 6 months; pilot RCT	[1] Control group	26 overweight or obese male and female with type 2 diabetes	[1] 49 ± 10.2	[1] 80	[1] 33.7 ± 2.7	*Weight change (%)* [1] 1.6 {−4.1, 3.8}		*HbA1c (%)* [1] 8.9 ± 1.6
	[2] Usual care and face-to-face group education and eHealth by app		[2] 59 ± 5.9	[2] 18	[2] 38.9 ± 9.0	[2] −1.8 {−4.2, −0.3}		[2] 6.9 ± 1.0
	[3] Usual care and face-to-face group education		[3] 56 ± 5.4	[3] 44	[3] 40.1 ± 7.0	[3] 0.4 {−2.3, 1.5}		[3] 9.1 ± 1.8 [D] **
Yin et al., 2022 [27] 6 months RCT	[1] Control group	120 overweight or obese male and female with type 2 diabetes	[1] 47 (42, 51)	[1] 38	[1] 29.05 (3.31)	*BMI (kg/m^2^*) [1] −1.68 (3.95)		*HbA1c (%)* [1] −1.84 (1.55) [2] −2.41 (1.38)
	[2] eHealth by app		[2] 48 (43, 51)	[2] 43	[2] 29.25 (2.93)	[2] −3.77 (3.38) **		*Fasting glucose (mmol/L)* [1] −2.83 (2.03) [2] −2.74 (1.96)

[1] = control group. [2] = intervention group 2. [3] = intervention group 3. [4] = intervention group 4. [A] = all groups. [D] = difference between groups. WC = waist circumference. Data depicted as: mean ± standard deviation (SD), mean (Standard Error (SE)), mean (95% Confidence interval (CI)), median {inter quartile range (IQR)}. * See supplementary material for explanation of interventions used. ** Between-group difference is significant (*p* < 0.05). # Significance not stated.

**Table 3:** Risk of bias summary.

Study ID	Randomissation Process	Deviations from Intended Intervention	Missing Outcome Data	Measurement of the Outcome	Selection of Reported Result	Overall
Castelnuovo	+	+	+	+	!	!
Dawes	!	+	+	+	+	+
Fischer	+	+	+	+	+	+
Haste	+	!	-	+	+	-
Lutes	+	+	+	+	!	+
Ma	+	+	+	+	+	+
Velasquez	!	+	+	+	!	!
Wang	+	+	+	+	+	+
Al Hamdan	-	-	+	+	+	-
Block	+	+	+	+	+	+
Katula	+	+	+	+	+	+
Lim	!	+	+	+	+	!
St Jules	+	+	+	+	+	+
Toro Ramos	+	+	+	+	+	+
Yin	+	-	+	+	+	-

The “-” in red color means high risk of bias; the “!” in yellow color means low risk of bias; the “+” in green color means no risk of bias.

## Data Availability

Data sharing is not applicable to this article.

## Supplementary Materials

The following are available online at https://www.mdpi.com/article/10.3390/nu15173776/s1, Table S1: Summary of search strategy. Table S2: Intervention description.
