# Supplementary material for: The Effect of Including eHealth in Dietary Interventions for Patients with Type 2 Diabetes with Overweight or Obesity: A Systematic Review
Source: Nutrients. 2023 Aug 29;15(17):3776. doi: 10.3390/nu15173776 (PMC10490324; doi:10.3390/nu15173776)
Supplement: Supplementary file 1 [file nutrients-15-03776-s001.zip › nutrients-2544510-supplementary.pdf]

Supplementary Materials Search strategy

Databased searched	Records	Records after duplicates removed
Embase.com (1971-)	1737	1029
Medline ALL Ovid (1946-)	1897	1885
Web of Science Core Collection (1975-)	2192	704
PSycINFO Ovid (1806-)	223	33
Cochrane CENTRAL register of trials (1992-)	607	83
**Total**	**6656**	**3734**

Table S1 Summary of search strategy

Embase.com (1971-) 1325

('diabetes mellitus'/exp OR 'diabetic patient'/exp OR 'diabetes education'/de OR (diabet*):ab,ti) AND ('obesity'/exp OR 'body weight loss'/exp OR 'weight loss program'/de OR 'diet therapy'/de OR 'diet restriction'/exp OR 'low calorie diet'/exp OR 'low fat diet'/exp OR (obes* OR overweight* OR adiposit* OR metabolic-syndrome* OR (weight NEXT/1 (loss OR losing OR management OR reduc* OR watch*)) OR (diet NEAR/3 (therap* OR restrict* OR low*-calor* OR low*-fat OR intervention*))):ab,ti) AND ('group education'/de OR 'group therapy'/exp OR 'workshop'/de OR ('peer group'/de AND (education/exp OR 'telehealth'/exp)) OR 'support group'/exp OR (((group OR group-based) NEXT/3 (educat* OR therap* OR motivational* OR intervention* OR treatment* OR diet* OR nutrition* OR session*)) OR workshop OR education-class* OR lifestyle-class* OR life-style-class* OR peer-education* OR ((support OR education*) NEXT/2 group*) OR support-group*):ab,ti) NOT ([Conference Abstract]/lim) NOT ((animal/exp OR animal*:de OR nonhuman/de) NOT ('human'/exp)) AND [English]/lim

Medline ALL Ovid (1946-) 1507

(exp Diabetes Mellitus/ OR (diabet*).ab,ti.) AND (exp Obesity/ OR Weight Loss/ OR Weight Reduction Programs/ OR Diet Therapy/ OR Diet Therapy.fs. OR Diet, Reducing/ OR Caloric Restriction/ OR Diet, Fat-Restricted/ OR (obes* OR overweight* OR adiposit* OR metabolic-syndrome* OR (weight ADJ (loss OR losing OR management OR reduc* OR watch*)) OR (diet ADJ3 (therap* OR restrict* OR low*-calor* OR low*-fat OR intervention*))).ab,ti.) AND (Psychotherapy, Group/ OR (Peer Group/ AND (Education/ OR Telemedicine/)) OR Self-Help Groups/ OR (((group) ADJ1 (educat* OR therap* OR motivational* OR intervention* OR treatment* OR diet* OR nutrition* OR session*)) OR ((group-based) ADJ3 (educat* OR therap* OR motivational* OR intervention* OR treatment* OR diet* OR nutrition* OR session*)) OR workshop OR education-class* OR lifestyle-class* OR life-style-class* OR peer-education* OR ((support OR education*) ADJ2 group*) OR support-group*).ab,ti.) NOT (news OR congres* OR abstract* OR book* OR chapter* OR dissertation abstract*).pt. NOT ((animal/ OR animal*:de OR nonhuman/) NOT (human/)) AND english.la.

PSycINFO Ovid (1806-) 195

(exp Diabetes Mellitus/ OR (diabet*).ab,ti.) AND (exp Obesity/ OR Weight Loss/ OR Weight Control/ OR Diets/ OR (obes* OR overweight* OR adiposit* OR metabolic-syndrome* OR (weight ADJ (loss OR losing OR management OR reduc* OR watch*)) OR (diet ADJ3 (therap* OR restrict* OR low*-calor* OR low*-fat OR intervention*))).ab,ti.) AND (Group Psychotherapy / OR Group Counseling / OR (Peers/ AND (Telemedicine/)) OR Support Groups / OR (((group) ADJ1 (educat* OR therap* OR motivational* OR intervention* OR treatment* OR diet* OR nutrition* OR

session*)) OR ((group-based) ADJ3 (educat* OR therap* OR motivational* OR intervention* OR treatment* OR diet* OR nutrition* OR session*)) OR workshop OR education-class* OR lifestyle-class* OR life-style-class* OR peer-education* OR ((support OR education*) ADJ2 group*) OR support-group*).ab,ti.) NOT (news OR congres* OR abstract* OR book* OR chapter* OR dissertation abstract*).pt. NOT ((animal.po. OR exp animals/) NOT human.po.) AND english.la.

Web of Science Core Collection (1975-) 696

TS=(((diabet*)) AND ((obes* OR overweight* OR adiposit* OR metabolic-syndrome* OR (weight NEAR/1 (loss OR losing OR management OR reduc* OR watch*)) OR (diet NEAR/2 (therap* OR restrict* OR low*-calor* OR low*-fat OR intervention*)))) AND ((((group) NEAR/0 (educat* OR therap* OR motivational* OR intervention* OR treatment* OR diet* OR nutrition* OR session*)) OR ((group-based) NEAR/2 (educat* OR therap* OR motivational* OR intervention* OR treatment* OR diet* OR nutrition* OR session*)) OR workshop OR education-class* OR lifestyle-class* OR life-style-class* OR peer-education* OR ((support OR education*) NEAR/2 group*) OR support-group*)) NOT ((animal* OR rat OR rats OR mouse OR mice OR murine*) NOT (human*))) AND DT=(article) AND LA=(english)

Cochrane CENTRAL register of trials (1992-) 444

((diabet*):ab,ti) AND ((obes* OR overweight* OR adiposit* OR metabolic-syndrome* OR (weight NEXT/1 (loss OR losing OR management OR reduc* OR watch*)) OR (diet NEAR/3 (therap* OR restrict* OR "low*-calor*" OR "low*-fat" OR intervention*))):ab,ti) AND ((((group) NEXT (educat* OR therap* OR motivational* OR intervention* OR treatment* OR diet* OR nutrition* OR session*)) OR ((group-based)

NEAR/3 (educat* OR therap* OR motivational* OR intervention* OR treatment* OR diet* OR nutrition* OR session*)) OR workshop OR education-class* OR "lifestyle-class*" OR "life-style-class*" OR peer-education* OR ((support OR education*) NEXT/2 group*) OR support-group*):ab,ti)

(aanvullend niet uitwerken tot SR)

('diabetes mellitus'/exp OR 'diabetic patient'/exp OR 'diabetes education'/de OR (diabet*):ab,ti) AND ('obesity'/exp OR 'body weight loss'/exp OR 'weight loss program'/de OR 'diet therapy'/de OR 'diet restriction'/exp OR 'low calorie diet'/exp OR 'low fat diet'/exp OR (obes* OR overweight* OR adiposit* OR metabolic-syndrome* OR (weight NEXT/1 (loss OR losing OR management OR reduc* OR watch*)) OR (diet NEAR/3 (therap* OR restrict* OR low*-calor* OR low*-fat OR intervention*))):ab,ti) AND ('telehealth'/exp OR 'mobile application'/de OR 'smartphone'/exp OR (e-health OR m-health OR telehealth OR ehealth OR tele-health OR mobile-app* OR smartphone*):ab,ti) NOT ([animals]/lim NOT [humans]/lim)

Supplementary Materials Intervention description

Study	Control group [1]	eHealth group [2]	Third group [3]	Fourth group [4]
Al Hamdan	Participants were given face to face lifestyle advice as standard, non-personalized counselling by the assigned primary healthcare centre physician, and interviewed only at baseline and after every 3 months at the primary healthcare centre. This advice included distribution of translated pamphlets and booklets with information related to lifestyle changes from proven effective programs.	Whatsapp program (WEP): Participants were provided with the same information as the GEP group, but the content was delivered through social media. In addition, the WEP participants were asked to download "Al-Nahdi Mobile App" an app-based educational program about lifestyle modifications emphasizing the importance of weight loss, healthy diet and physical activity. WhatsApp messages were sent to the group by the	Group intensive lifestyle education program (GEP): Participants received structured group educational instructions and follow-up support for lifestyle modification from the study dietitian at least six times (at baseline and every 2 weeks during the first 3-month intervention period. Participants attended a total of six sessions covering the following topics: (i) prediabetes, risk of type 2 diabetes and setting achievable goals; (ii) nutrition	-

Study	Control group [1]	eHealth group [2]	Third group [3]	Fourth group [4]
		diabetes educator, and the participants were provided with a member card to join the gym. The contents of these messages were about diet therapy, exercise, general diabetes and prediabetes care knowledge. Participants were provided with Accucheck softclix lancing device for blood sugar monitoring purposes at any time during the study.	for health and weight management; (iii) carbohydrate awareness; (iv) reading and understanding food labels; (v) exercise and the benefits of physical activity; and (vi) healthcare essentials that include monitoring and a checklist. All presentations were delivered by certified personnel (dietitian and diabetes educator).	
Block	Brief instruction that they were at risk for developing diabetes and that increased physical activity as well as changes in their dietary	Alive-PD is a fully automated intervention, with no human coaching or advice. It is delivered via an individualized website	-	-

Study	Control group [1]	eHealth group [2]	Third group [3]	Fourth group [4]
	behaviours could help prevent progression to diabetes.	and interactive emails and is supplemented by a mobile app and automated phone and print modules. Participants complete a diet and physical activity assessment online at baseline, followed by immediate automated individualized feedback. They also complete an activity planning tool to guide improvements in aerobic activity. Participants then engage in a program of weekly tailored goal setting and tracking over the next 6 months and every other week		

Study	Control group [1]	eHealth group [2]	Third group [3]	Fourth group [4]
		for the subsequent 6 months. Participants work simultaneously on dietary and physical activity behaviours. Participants are invited to choose a long-term weight loss target of 5%, 7%, or 10% of bodyweight. The focus throughout is on the objective of lowering haemoglobin A1c and preventing diabetes.		
Castelnuovo	One-month inpatient intervention for weight loss and medical care. No other intervention was offered.	Tecnob group: One-month inpatient intervention for weight loss and medical care. Patients are instructed to measure energy expenditure and	-	-

Study	Control group [1]	eHealth group [2]	Third group [3]	Fourth group [4]
		physical activity (duration and levels) with a multi-sensory armband 36 hours every 2 weeks and upload information so they can view their progress. Furthermore, they are instructed to use the interactive Tecnob website or Software on their mobile phone. This website or software contains questionnaires, food diary, videoconferencing calls with clinician and contact options with the dietician.		
Dawes	Usual care from their general practitioner.	Flip intervention:<br>Lifestyle prescription (brief advice and statement of	-	-

Study	Control group [1]	eHealth group [2]	Third group [3]	Fourth group [4]
		intent to modify health behaviour, including long term goals by healthy eating, weight, and physical activity), pedometer (used to increase physical activity), support by community-based facilitator (telephone call every 2 months).		
Fischer	All standard of care weight loss resources could be used.	All standard of care weight loss resources could be used. Furthermore, they received text messages 6 times per week based on Diabetes Prevention Program (DPP)* subjects. Also, motivational interviews with a health coach were available.	-	-

Study	Control group [1]	eHealth group [2]	Third group [3]	Fourth group [4]
Haste	Usual care.	My dietician website: Individual dietary advice monitored through web consultations with a dietician. Website also contained options to record food diary, physical activity, weight. Patients were advised to do this daily.	-	
Katula	Enhanced standard care: Consisted of a 1-time, 2-hour diabetes prevention education class consisting of 12−18 participants, led by a health educator or graduate student with training in nutrition, physical activity, and diabetes prevention	The digital-DPP: Includes an initial 16-week intensive curriculum focusing on weight loss, followed by a 36-week curriculum focusing on weight maintenance, with a total of 12 months of novel lessons. Using internet-enabled devices, participants	-	-

Study	Control group [1]	eHealth group [2]	Third group [3]	Fourth group [4]
	strategies. The class included detailed information on current recommendations for physical activity and healthy food choices involving portion size, eating regular meals, and a well-balanced diet based on My Plate recommendations and the development of a personal action plan to prevent diabetes.	asynchronously completed the weekly, interactive behaviour change curriculum lessons; engaged in private, asynchronous messages with a trained lifestyle health coach; engaged in asynchronous discussions with a virtual peer group; tracked meals; monitored weight with a wireless scale; and tracked physical activity with connected wearable devices. Health coaches facilitated group interaction by group discussion boards, contacting participants through secure messaging to		

Study	Control group [1]	eHealth group [2]	Third group [3]	Fourth group [4]
		provide feedback and advice during key and reinforcing lesson content.		
Lim	Received a single 45- to 60-minute advisory session from a registered research dietitian concerning diet and physical activity, as per American Dietetic Association (ADA) guidelines at baseline and continued to receive standard diabetes care from their usual health care professionals.	Received a single 45- to 60-minute advisory session from a registered research dietitian concerning diet and physical activity, as per ADA guidelines at baseline and continued to receive standard diabetes care from their usual health care professionals. Additionally the intervention group was required to use the app for 6 months to track weight twice weekly and diet and physical activity daily, and to	-	-

Study	Control group [1]	eHealth group [2]	Third group [3]	Fourth group [4]
		communicate regularly with the research dietitians via the app. Intervention participants chose a weight loss goal of 3%to 10%, depending on individual preferences, and were encouraged to achieve individualized calorie and carbohydrate goals and an activity goal of 10 000 steps daily set by the app. They were also provided with a glucometer to track fasting and postprandial blood glucose 2 days weekly. Educational videos were pushed to the participants weekly via the app in the first		

Study	Control group [1]	eHealth group [2]	Third group [3]	Fourth group [4]
		3 months. Dietitian supported the participants by messaging them via the app every few days in the first 3 months, and weekly in the subsequent 3 months, spending 1 to 15 minutes on each participant each time. They regularly reviewed goals with intervention participants, provided individualized feedback, including helping them to identify and cope with barriers and to use prompts and cues.		
Lutes	16-week EMPOWER manual with recording forms, weight	Six emails containing diet selection, healthy snacking,	-	-

Study	Control group [1]	eHealth group [2]	Third group [3]	Fourth group [4]
	scale, glucose monitor, pedometer. Patients had a session with a community health worker every week where they discussed self-monitoring, goal setting, nutrition, physical activity, skill power versus willpower, diabetes 101, planning and time management, communication, mindfulness, and awareness, breaking negative thought chains, dealing with slips/challenges, coping with stress, utilizing the community, and problem solving.	managing medications, monitoring blood glucose, and engaging in physical activity.		
Ma	Standard medical care.	Self-directed:	Coach led:	-

Study	Control group [1]	eHealth group [2]	Third group [3]	Fourth group [4]
	No information about weight loss or weight-loss goals.	12-session DPP lifestyle intervention curriculum, Group Lifestyle Balance. Via a home-based DVD. Also, they had one class in which they were trained to use the AHA free Heart360 web portal for weight and physical activity goal setting and self-monitoring and were given a weight scale and pedometer. Furthermore, biweekly reminder messages about self-monitoring throughout the intensive and maintenance phase were sent, and monthly motivational messages were	12-session DPP lifestyle intervention curriculum, face-to-face in 12-weekly classes, class one they were trained to use the AHA free Heart360 web portal for weight and physical activity goal setting and self-monitoring and were given a weight scale and pedometer. In addition, there were food tastings at check-in and 30 to 45 minutes of guided physical activity at the end of each weekly class. Furthermore, monthly motivational messages were sent during the maintenance phase and personalized	

Study	Control group [1]	eHealth group [2]	Third group [3]	Fourth group [4]
		sent during this phase.	messages on at least a monthly basis were send in intensive and maintenance face.	
St-Jules	Advice:<br>Participants continued to receive routine care and were provided the same prescription for diet and physical activity. Participants received written information about their intervention targets through US mail, which were then reviewed for clarity via a follow-up telephone call by the study registered dietitian. During the subsequent intervention	Monitoring:<br>Participants continued to receive routine care and were provided the same prescription for diet and physical activity. Participants were provided with a tablet and prepaid 4G data plan to use during the study. Tablets were preloaded with WebEx for videoconferencing and MyNetDiary for self-monitoring, as appropriate for their randomization	SCT:<br>Participants continued to receive routine care and were provided the same prescription for diet and physical activity. Participants were provided with a Tablet and prepaid 4G data plan to use during the study. Tablets were preloaded with WebEx for videoconferencing and MyNetDiary for self-monitoring, as appropriate for their randomization	Combined:<br>Participants continued to receive routine care and were provided the same prescription for diet and physical activity. Participants were provided with a Tablet and prepaid 4G data plan to use during the study. Tablets were preloaded with WebEx for videoconferencing and MyNetDiary for self-monitoring, as appropriate for their randomization

Study	Control group [1]	eHealth group [2]	Third group [3]	Fourth group [4]
	period, ADVICE participants were mailed monthly 1-page educational handouts outlining the rationale and recommendations for achieving said intervention targets.	assignment. Participants received in-person, one-on-one training in the use of the iPads and relevant software applications lasting approximately 1 hour. Participants were provided group-based WebEx videoconferencing sessions led by the study registered dietitian. Sessions were delivered every week for the first 4 weeks, then every other week up to 20 weeks (14 sessions total). Group sessions were anchored by Microsoft PowerPoint presentations which were	assignment. Participants received in-person, one-on-one training in the use of the iPads and relevant software applications lasting approximately 1 hour. Participants were provided group-based WebEx videoconferencing sessions led by the study registered dietitian. Sessions were delivered at a frequency of every week for the first 4 weeks, then every other week up to 20 weeks (14 sessions total). Group sessions were anchored by Microsoft PowerPoint	assignment. Participants received in-person, one-on-one training in the use of the tablets and relevant software applications lasting approximately 1 hour. Participants were provided group-based WebEx videoconferencing sessions led by the study registered dietitian. Sessions were delivered at a frequency of every week for the first 4 weeks, then every other week up to 20 weeks (14 sessions total). Group sessions were anchored by Microsoft PowerPoint

Study	Control group [1]	eHealth group [2]	Third group [3]	Fourth group [4]
		educational. Participants were also provided a 6-month subscriptions to MyNetDiary food logging mobile application, and directed to record all food and beverage intake, as well as their physical activity and body weight (weekly) into the program. After each group session, participants received personalized feedback reports on self-monitoring, diet, physical activity, and weight via email from the study registered dietitian.	presentations which were which were educational and behavioural.	presentations which were which were educational and behavioural. Participants were also provided a 6-month subscriptions to MyNetDiary food logging mobile application, and directed to record all food and beverage intake, as well as their physical activity and body weight (weekly) into the program. After each group session, participants received personalized feedback reports on self-monitoring, diet, physical activity, and weight via email from the study registered dietitian.

Study	Control group [1]	eHealth group [2]	Third group [3]	Fourth group [4]
Toro-Ramos	Usual care and printed version of the DPP curriculum.	Participants learned how to use the Noom program, how to interact with their coach, and the importance of maintaining motivation throughout the program. Participants had mobile access to coach-participant messaging, group messaging, daily challenges for behaviour change, the DPP education articles, food logging with colour coding, steps and exercise logging, and automated feedback based on food choices. They were asked to log their weight by self-report, meals,	-	-

Study	Control group [1]	eHealth group [2]	Third group [3]	Fourth group [4]
		and physical activity within the program on a weekly basis. Coaches securely monitored participant progress through a web-based dashboard. Participants could communicate as needed to support their individual journeys and could expect to hear from their coach every day.		
Velasquez	Personalized nutritional therapy from a certified nutritionist. Based on a three-day food intake recording and a 24h recall. The nutritional recommendations	Personalized nutritional therapy from a certified nutritionist. Based on a three-day food intake recording and a 24h recall. The nutritional recommendations	-	-

Study	Control group [1]	eHealth group [2]	Third group [3]	Fourth group [4]
	were assigned in accordance with the Mexican and the American Diabetes Association Guidelines for Diabetes. The prescribed diet with five sample menus were given including a section for recording tips and healthy diet and physical exercise recommendations.	were assigned in accordance with the Mexican and the American Diabetes Association Guidelines for Diabetes. And they got assess to the multimedia program Nutriluv® with seven modules with information on diabetes, nutrition, and healthy lifestyle. The educational tool was used before every visit.		
Wang	Usual care and diabetes education from their primary care physicians and diabetes educators according to a diabetes education program.	Usual care and diabetes education from their primary care physicians and diabetes educators according to a diabetes education program. This group used the LoseIt!	Usual care and diabetes education from their primary care physicians and diabetes educators according to a diabetes education program. This group monitored	-

Study	Control group [1]	eHealth group [2]	Third group [3]	Fourth group [4]
		app for monitoring of diet, exercise, and weight. They used the Diabetes Connect app to monitor blood glucose levels. There were no prompts or reminders used in these apps. In addition, a standard behavioural lifestyle intervention comprising 11 group sessions were offered according to the DPP program. And participants were trained on self-monitoring their diet, exercise, weight, and blood glucose.	themselves on paper journals. In addition, a standard behavioural lifestyle intervention comprising 11 group sessions were offered according to the DPP program. And participants were trained on self-monitoring their diet, exercise, weight, and blood glucose.	
Yin	Patients were followed up through conventional	Postprandial blood glucose (PBG) and fasting blood	-	-

Study	Control group [1]	eHealth group [2]	Third group [3]	Fourth group [4]
	outpatient clinic appointments every 2 weeks, and telephone follow-up was used during the isolation period. The participants received traditional health education, which included diet, exercise, and medication guidance, during clinic visits.	glucose (FBG) levels were monitored using a glucometer. Patients were provided training for independently using the hospital's telemedicine app. The glucometer was connected to the patient's mobile phone via Bluetooth. The glucometer data were then automatically transferred to the hospital telemedicine app. The patients were followed up four times a week in the first 3 months and twice a week in the next 3 months. Doctors reminded patients to monitor		

Study	Control group [1]	eHealth group [2]	Third group [3]	Fourth group [4]
		their blood glucose levels and provided medical advice through the telemedicine system. the dietitian advised the patients on energy intake and food exchange methods. They were provided custom-tailored dietary recommendations and were asked to consume the required calories and upload their daily dietary intake on the telemedicine app. Additionally, the app recorded the patients' daily steps and automatically transferred them to the medical server.		

Table S2 Intervention description

*DPP = a lifestyle program which covers the following subjects: Diet (Eat Less Fat and Fewer Calories, Healthy Eating), exercise (Move Those Muscles, Being Active - A way of Life, Take Charge of What's Around You) and Problem Solving (Four Keys to Healthy Eating Out, Talk Back to Negative Thoughts, The Slippery Slope of Lifestyle Change, Make Social Cues Work for You, You Can Manage Stress, Ways to Stay Motivated).
